# Noninvasive prediction of classic EGFR-activating mutations in non-small cell lung cancer using a combined spectral computed tomography model: an auxiliary tool for preoperative stratification of candidates for EGFR-TKI therapy

**DOI:** 10.1186/s12890-026-04575-7

**Published:** 2026-08-11

**Authors:** Qianqian Zhan, Hui Lin, Dandan Lin, Xueyan Liao

**Affiliations:** Department of Radiology, Longyan First Affiliated Hospital of Fujian Medical University, Longyan, Fujian Province China

**Keywords:** EGFR mutation, Non-small cell lung cancer, Spectral CT, Preoperative prediction, EGFR-TKI

## Abstract

**Background:**

EGFR genotyping is essential for EGFR-TKI therapy in non-small cell lung cancer (NSCLC), but invasive biopsy has limitations. Noninvasive preoperative stratification tools are urgently needed.

**Methods:**

We retrospectively enrolled 129 NSCLC patients to develop and internally validate a combined model integrating clinical features, conventional CT findings, and dual-layer spectral CT parameters for predicting classic EGFR-activating mutations. Performance was evaluated by ROC, DeLong test, DCA, and 1000-iteration bootstrap validation.

**Results:**

The combined model achieved an AUC of 0.805, significantly outperforming the clinical-conventional CT model (AUC = 0.642, *P* < 0.001). Venous-phase normalized iodine concentration (NIC) was the only independent predictor (OR = 2.578, *P* = 0.018). The model showed robust performance in the lung adenocarcinoma subgroup (AUC = 0.810) with negligible overfitting.

**Conclusions:**

This noninvasive spectral CT model provides a reliable, easy-to-implement auxiliary tool for preoperative EGFR mutation stratification to optimize EGFR-TKI therapy decisions.

**Supplementary Information:**

The online version contains supplementary material available at https://doi.org/10.1186/s12890-026-04575-7.

## Highlight

Background: Epidermal growth factor receptor (EGFR) genotyping is essential for EGFR‑tyrosine kinase inhibitor (TKI) therapy in non‑small cell lung cancer (NSCLC), but invasive biopsy has limitations. Noninvasive predictive tools are urgently needed for preoperative stratification.

Methods: We developed and internally validated a combined model using clinical features, conventional CT findings, and dual‑layer spectral CT parameters to predict classic EGFR‑activating mutations in 129 NSCLC patients.

Results: The combined model achieved an AUC of 0.805, significantly outperforming the clinical‑conventional CT model. Venous‑phase normalized iodine concentration (NIC) was the only independent predictor.

Conclusions: This noninvasive spectral CT model provides a robust, easy‑to‑implement auxiliary tool for preoperative EGFR mutation stratification to optimize EGFR‑TKI therapy decisions.

**Supplementary Information:**

The online version contains supplementary material available at https://doi.org/10.1186/s12890-026-04575-7.

## Introduction

Lung cancer remains the leading cause of cancer-related mortality worldwide, with non-small cell lung cancer (NSCLC) accounting for approximately 85% of cases [1, 2]. EGFR tyrosine kinase inhibitors (TKIs) have revolutionized NSCLC management, and first-line EGFR-TKIs provide significant survival and quality-of-life benefits over chemotherapy in patients with classic EGFR-activating mutations [3]. The IPASS trial first established EGFR genotyping as the cornerstone of TKI decision-making [4]. The ADAURA trial further demonstrated the disease-free survival benefit of adjuvant osimertinib in resectable EGFR-mutant NSCLC [5], creating an urgent need for rapid, noninvasive preoperative EGFR stratification to optimize perioperative TKI therapy.

However, tissue-based EGFR genotyping is limited by invasiveness, procedural risks, and turnaround time, and liquid biopsy using ctDNA still faces technical and biological constraints [6]. Although conventional CT is routinely performed for preoperative staging, it only provides morphological characterization with limited insight into tumor molecular features. Dual-layer spectral CT offers quantitative parameters—notably normalized iodine concentration (NIC) and spectral curve slope (λHU)—that reflect tumor perfusion and microvascular density, showing promise for evaluating tumor biological behavior [7]. While previous studies have explored spectral CT parameters for EGFR mutation prediction [8], comprehensive models integrating clinical, conventional CT, and multiphasic spectral CT data remain scarce, particularly regarding their utility in EGFR-TKI workflows. While previous studies including Jin et al. [9] have explored spectral CT-based radiomics models, such approaches require specialized post-processing and complex feature extraction, limiting widespread implementation. In contrast, our model uses standardized, readily obtainable spectral CT parameters that can be seamlessly integrated into routine workflows without additional software or dedicated training.

Therefore, this study aimed to develop and internally validate a noninvasive combined model integrating clinical features, conventional CT findings, and dual-phase spectral CT parameters for preoperative prediction of classic EGFR-activating mutations (exon 19 deletions and exon 21 L858R) in NSCLC. This model is intended as a readily implementable adjunct to tissue genotyping to streamline preoperative patient stratification for first-line and adjuvant EGFR-TKI therapy.

## Materials and methods

### Study population

This retrospective study consecutively screened 221 patients with pathologically confirmed NSCLC at Longyan First Affiliated Hospital of Fujian Medical University between August 2023 and October 2025. We excluded patients according to the pre-specified criteria: 32 patients without standardized preoperative dual-layer spectral CT examination in our hospital; 24 patients who received anticancer treatment (chemotherapy, targeted therapy, radiotherapy, etc.) before CT examination; 17 patients with pulmonary nodules < 1 cm in maximum diameter or subsolid nodules dominated by ground-glass opacity; 10 patients with incomplete clinical, imaging or molecular testing data; 5 patients without valid EGFR genotyping results; 4 patients carrying rare EGFR mutations. Finally, a total of 129 eligible patients were enrolled in this study, and were stratified into EGFR mutation-positive group (*n* = 63) and wild-type group (*n* = 66) based on tissue molecular testing results.

All enrolled patients underwent preoperative spectral CT examination within 2 weeks before surgical resection or biopsy, to ensure the consistency between imaging findings and pathological/molecular testing results. There were 88 male and 41 female patients, with a mean age of 65.28 ± 10.21 years (range, 32–89 years). Only adenocarcinoma and squamous cell carcinoma were included in this cohort, which constitute the vast majority of NSCLC cases, ensuring the broad clinical representativeness of our study population.

#### Inclusion criteria

1. Histopathologically confirmed NSCLC;
2. Available EGFR genotyping results of tumor tissue;
3. No prior anticancer treatment before spectral CT examination;
4. Single solid pulmonary nodule or mass > 1 cm in maximum diameter.

#### Exclusion criteria

1. Incomplete clinical or imaging data;
2. Subsolid nodules predominantly consisting of ground-glass opacity;
3. Absence of valid EGFR mutation testing results;
4. Patients with rare EGFR mutations (such as exon 20 insertion, exon 18 G719X, etc.).

For patients with multiple pulmonary nodules, only the pathologically confirmed primary index lesion was subjected to radiological subtyping and quantitative analysis; non-target nodules did not affect study eligibility. Sample size adequacy was evaluated according to the widely accepted 10 events per variable (EPV) rule for multivariate logistic regression. With 6 predictor variables in the final model and 63 EGFR mutation-positive cases, the EPV was 10.5, meeting the recommended threshold of ≥ 10 and supporting adequate statistical power for model construction and internal validation. This sample size justification aligns with the TRIPOD reporting guidelines for prediction model studies [10].

### EGFR genotyping method

EGFR mutation status was detected using amplification refractory mutation system polymerase chain reaction (ARMS-PCR). Formalin-fixed paraffin-embedded (FFPE) tissue specimens were obtained from surgical resection, percutaneous needle biopsy, or bronchoscopic biopsy of enrolled patients. All assays were performed in the Department of Pathology of Longyan First Affiliated Hospital of Fujian Medical University, Longyan, Fujian Province, China, strictly following the manufacturer’s protocol of the 5 Mutations Gene Detection Kit (Fluorescence PCR Method, Amoy Diagnostics Co., Ltd., Xiamen, Fujian Province, China).

The detection panel covered classic EGFR-activating mutations, including exon 19 deletions and exon 21 L858R point mutations. Rare EGFR alterations (e.g., exon 20 insertions) were excluded from the mutation-positive group, since they show inconsistent or diminished sensitivity to first-line EGFR-TKIs compared with classic mutations [11]. This strict definition of classic EGFR-activating mutation ensures that the model specifically predicts patients eligible for currently approved EGFR-TKI therapy, greatly enhancing the direct clinical relevance of our findings for precision oncology practice. ARMS-PCR is a routine clinical method for detecting classic EGFR-activating mutations in China and shows high concordance with NGS for exon 19 deletions and exon 21 L858R, supporting the reliability of the molecular testing results in this study.

### Ethics statement

This retrospective study was approved by the Institutional Review Board of Longyan First Affiliated Hospital of Fujian Medical University, Longyan, Fujian Province, China (Approval No. LYREC2026-k054-01), and the requirement for written informed consent was waived due to the retrospective nature of the study. All procedures were performed in accordance with the Declaration of Helsinki.

### Spectral CT acquisition

All CT examinations were performed on a Philips IQon dual-layer detector spectral CT scanner (Philips Healthcare, Best, the Netherlands). After breath-hold training, unenhanced chest CT was performed from the thoracic inlet to the lung base, followed by dual-phase contrast-enhanced CT in a single spectral acquisition mode.

Iodinated contrast material (iohexol, OMNIPAQUE^®^, 300 mgI/mL, GE Healthcare, Shanghai, China) was injected intravenously at a flow rate of 2.5–3.0 mL/s, with total contrast volume calculated as 1.5 mL/kg of body weight (maximum 80 mL), followed by a 30-mL saline flush. The arterial phase was automatically triggered at a threshold of 100 HU in the descending aorta, and the venous phase was acquired at 60–70 s after contrast administration.

Scanning parameters were as follows: 120 kVp, automated tube current modulation, collimation of 64 × 0.625 mm, rotation time of 0.5 s, field of view of 500 mm, and pitch of 1.015. All images were reconstructed with iDose4 iterative reconstruction at 1-mm section thickness and 1-mm interval, using a standard soft-tissue kernel.

### Image analysis and quantitative measurements

All image reconstructions and quantitative analyses were performed on a Philips IntelliSpace Portal workstation using the dedicated spectral imaging package. Two radiologists with at least 5 years of experience in thoracic imaging independently performed quantitative measurements on 30 randomly selected cases (23% of the total cohort) in a blinded manner to clinical, pathological, and EGFR mutation status, to verify the reliability of the measurement method. Inter-observer agreement for all core spectral CT quantitative parameters was assessed using a two-way mixed-effects model, absolute agreement, single-measure ICC. An ICC value > 0.80 was defined as excellent agreement.

After confirming excellent inter-observer agreement, all quantitative measurements for the entire cohort were performed by a senior radiologist with 8 years of thoracic imaging experience, and independently verified by another radiologist with 5 years of experience. Discrepancies were resolved by consensus discussion.

Circular or oval regions of interest (ROIs) were placed on the largest solid and homogeneous component of the tumor, avoiding visible vessels, calcifications, necrosis, hemorrhage, and adjacent atelectasis. The ROI size was adjusted according to the lesion dimension and kept consistent by using the copy function for all measurements. All parameters were measured in triplicate, and the mean values were used for subsequent analysis. The 2D ROI delineation protocol used in this study aligns better with routine clinical workflow and allows rapid measurement, balancing predictive accuracy and clinical practicality.

### Conventional CT findings

Maximum tumor diameter, spiculation, lobulation, pleural indentation, and bronchial cutoff signs were recorded.

### Spectral CT quantitative parameters

The following quantitative parameters were obtained in both arterial and venous phases:

- 70-keV monoenergetic CT value (CT70keV), Effective atomic number (Z-eff), Iodine concentration (IC), Spectral curve slope (λHU).

NIC was calculated as the ratio of tumor IC to the IC of the descending aorta at the same slice level, using an identical-sized ROI placed in the lumen of the descending aorta:

- Arterial-phase NIC (NICA) = Tumor IC (arterial phase) / Descending aorta IC (arterial phase)
- Venous-phase NIC (NICV) = Tumor IC (venous phase) / Descending aorta IC (venous phase)

λHU was calculated as:

- Arterial-phase λHU (λHUA) = (CT value at 40 keV − CT value at 100 keV) / 60 (arterial phase)
- Venous-phase λHU (λHUV) = (CT value at 40 keV − CT value at 100 keV) / 60 (venous phase)

### Tumor staging and clinical data collection

TNM staging was performed according to the 8th edition of the TNM staging system proposed by the International Association for the Study of Lung Cancer (IASLC) [12]. Clinical data including sex and pathological type were also recorded.

### Statistical analysis

All statistical analyses and model development were performed in accordance with the Transparent Reporting of a multivariable prediction model for Individual Prognosis Or Diagnosis (TRIPOD) guidelines. Statistical analyses were performed using SPSS 27.0 software (IBM Corp., Armonk, NY, USA). Categorical variables were presented as frequencies and percentages, and compared using the chi-square test. Continuous variables were tested for normality using the Kolmogorov–Smirnov test. Normally distributed data were expressed as mean ± standard deviation and compared using independent-samples t-tests. Non-normally distributed data were expressed as median (interquartile range) and compared using the Mann-Whitney U test. For large-sample demographic variables with mild skewness, t-test was applied per the central limit theorem.

Variables with statistically significant differences (*P* < 0.05) in univariate analysis were included in multivariate binary logistic regression analysis to construct predictive models for EGFR mutation status. For variables with P values close to the threshold, we did not include them in the final model based on clinical judgment, to control model complexity and reduce the risk of overfitting. Before multivariate logistic regression, multicollinearity among variables was tested using the variance inflation factor (VIF). The VIF values of all included variables ranged from 1.045 to 1.758, all far below the critical threshold of 5, indicating no significant multicollinearity, and all variables were retained in the final regression model.

ROC curves were generated, and predictive performance was assessed using the AUC, sensitivity, specificity, positive predictive value (PPV), negative predictive value (NPV), positive likelihood ratio (PLR), and negative likelihood ratio (NLR). The DeLong test was used to compare AUCs between different models, with a *P*-value < 0.05 considered statistically significant. The Hosmer-Lemeshow (H-L) test was used to evaluate the calibration of the models, with a *P*-value > 0.05 indicating good calibration. The optimal cut-off value was determined by the maximum Youden index.

The Brier score (range 0–1, lower values indicate higher prediction accuracy) was calculated to quantify the overall prediction error of the two models. DCA was performed to evaluate the clinical net benefit of the models across clinically relevant threshold probabilities, by comparing the net benefit of model-guided treatment decisions vs. the default strategies of treating all patients or treating none. Internal validation of the final combined prediction model was performed using the bootstrap resampling method with 1000 iterations of replacement sampling to correct for overfitting bias. The robustness of the model and the stability of the regression coefficients of each predictor were evaluated via bootstrap resampling. The bootstrap-corrected AUC and calibration slope were calculated to assess the model’s discrimination and overfitting degree. Pre-specified subgroup analysis was performed in patients with lung adenocarcinoma, the core target population for first-line EGFR-TKI therapy, to assess the model’s performance in this clinically relevant cohort. A two-sided *P*-value < 0.05 was considered statistically significant.There were no missing data in clinical, imaging, and molecular testing data for all enrolled patients, so no imputation was performed for missing data.

## Results

A total of 129 patients were included, of whom 63 (48.8%) harbored classic EGFR-activating mutations and 66 (51.2%) were wild-type.

### Inter-observer agreement

Two independent radiologists (with 5 and 8 years of thoracic imaging experience, respectively) performed blinded quantitative measurements on 30 randomly selected cases. Inter-observer agreement was good to excellent for all core quantitative spectral CT parameters, with intraclass correlation coefficients (ICC) of representative core parameters presented as follows: venous-phase NIC: 0.89 (95% CI: 0.69–0.95), venous-phase λHU: 0.93 ( 95%CI: 0.84–0.97), Arterial-phase λHU: 0.91 ( 95%CI: 0.83–0.96), venous-phase CT70keV: 0.90 (95% CI: 0.81–0.95). All ICC values ranged from 0.85 to 0.93 (all *P* < 0.001), confirming the high reliability and repeatability of our imaging measurement protocol, supporting the stability of the quantitative data used for model construction.

### Clinical and conventional CT findings

Adenocarcinoma histology was significantly more frequent in the EGFR mutation-positive group (82.5% vs. 66.7% in the wild-type group, *P* = 0.039), as was the presence of lobulation sign (71.4% vs. 51.5% in the wild-type group, *P* = 0.020). These findings are consistent with previous reports linking lobulation sign and adenocarcinoma histology to EGFR-mutant lung adenocarcinoma [13, 14]. There were no significant differences in sex, age, TNM stage, tumor size, spiculation, pleural indentation, or bronchial cutoff signs between groups (all *P* > 0.05) (Table 1).

**Table 1:** Clinical and conventional CT characteristics of the two groups

Characteristic	EGFR-positive (*n* = 63)	EGFR-negative (*n* = 66)	Statistic	*P*-value
Age (years)	63.67 ± 11.79	66.82 ± 8.24	*t* = 1.753	0.082
Male/female, n	38 / 25	50 / 16	χ² = 3.544	0.060
Pathological type (adeno/squamous)	52 / 11	44 / 22	χ² = 4.266	**0.039**
TNM stage (I–II / III–IV)	24 / 39	19 / 47	χ² = 1.256	0.262
Tumor max diameter (mm), Median(P25,P75)	30.00 (19.00, 48.00)	34.00 (25.50, 51.25)	*Z* = 1.343	0.179
Lobulation sign (yes/no)	45 / 18	34 / 32	χ² = 5.385	**0.020**
Spiculation sign (yes/no)	28 / 35	25 / 41	χ² = 0.574	0.449
Pleural indentation (yes/no)	18 / 45	12 / 54	χ² = 1.949	0.163
Bronchial cutoff (yes/no)	34 / 29	36 / 30	χ² = 0.004	0.948

*Abbreviations*: *EGFR* epidermal growth factor receptor, *TNM* tumor-node-metastasis. Data are presented as mean ± standard deviation, median (interquartile range), or n. *P*-values were calculated using independent-samples t-test, Mann-Whitney U test, or chi-square test. Bold values indicate statistical significance (*P* < 0.05)

### Spectral CT quantitative parameters

Arterial-phase λHU, venous-phase NIC, venous-phase λHU, and venous-phase CT70keV were significantly higher in the EGFR mutation-positive group (all *P* < 0.05) (Table 2). No significant differences were observed in arterial-phase NIC, arterial-phase CT70keV, or effective atomic number in either phase (all *P* > 0.05).

**Table 2:** Spectral CT quantitative parameters of the two groups

Parameter	EGFR-positive (*n* = 63)	EGFR-negative (*n* = 66)	t-value	*P*-value
Arterial-phase NIC	0.12 ± 0.04	0.11 ± 0.03	1.920	0.057
Venous-phase NIC	0.40 ± 0.06	0.36 ± 0.06	4.211	**< 0.001**
Arterial-phase λHU	1.28 ± 0.40	1.02 ± 0.35	3.945	**< 0.001**
Venous-phase λHU	1.72 ± 0.39	1.36 ± 0.42	5.018	**< 0.001**
Arterial-phase CT70keV (HU)	61.79 ± 14.90	58.86 ± 10.56	1.282	0.202
Venous-phase CT70keV (HU)	73.80 ± 14.31	66.79 ± 10.45	3.168	**0.002**
Effective atomic number (arterial)	7.82 ± 0.18	7.77 ± 0.19	1.410	0.161
Effective atomic number (venous)	7.97 ± 0.18	7.91 ± 0.18	1.970	0.051

*Abbreviations*: *NIC* normalized iodine concentration, *CT70keV* 70-keV monoenergetic CT value, *λHU* spectral curve slope, *HU* Hounsfield unit. Data are presented as mean ± standard deviation. *P*-values were calculated using independent-samples t-test. Bold values indicate statistical significance (*P* < 0.05)

### Performance of single spectral CT parameters

**Fig. 1:**
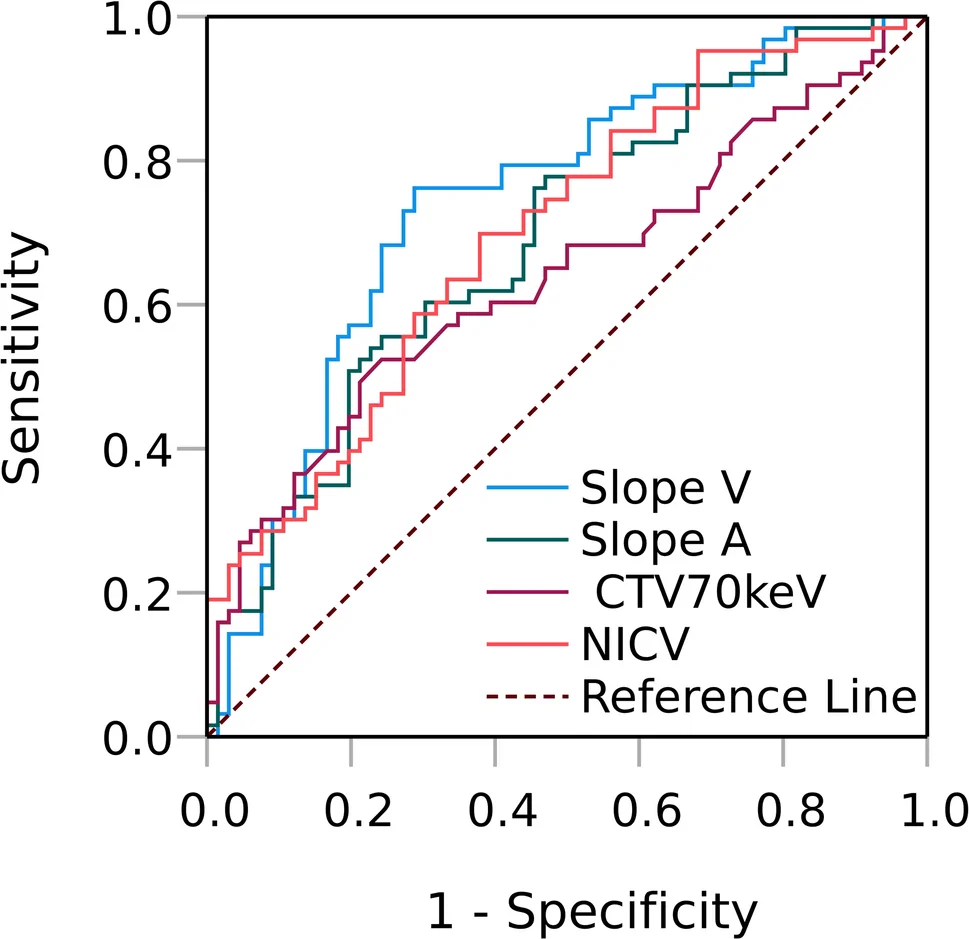
Receiver operating characteristic (ROC) curves of single spectral computed tomography (CT) parameters for predicting epidermal growth factor receptor (EGFR) mutation status in non-small cell lung cancer (NSCLC). The venous-phase spectral curve slope presents the highest predictive value with an area under the curve (AUC) of 0.741. Abbreviations: ROC, receiver operating characteristic; CT, computed tomography; EGFR, epidermal growth factor receptor; NSCLC, non-small cell lung cancer; AUC, area under the curve; NICV, venous-phase normalized iodine concentration; Slope A, arterial-phase spectral curve slope; Slope V, venous-phase spectral curve slope; CTV70keV, venous-phase 70-keV monoenergetic CT value

ROC analysis demonstrated that venous-phase λHU yielded the highest predictive efficacy for EGFR mutation, with an AUC of 0.741 (95% CI: 0.655–0.828), followed by venous-phase NIC (AUC = 0.700, 95% CI: 0.611–0.789) and venous-phase CT70keV (AUC = 0.641, 95% CI: 0.545–0.738). DeLong test confirmed that the AUC of venous-phase λHU was significantly higher than that of venous-phase CT70keV (*P* = 0.032), while no significant difference was observed between venous-phase λHU and venous-phase NIC (*P* = 0.317) (Fig. 1).

### Multivariate logistic regression analysis

**Fig. 2:**
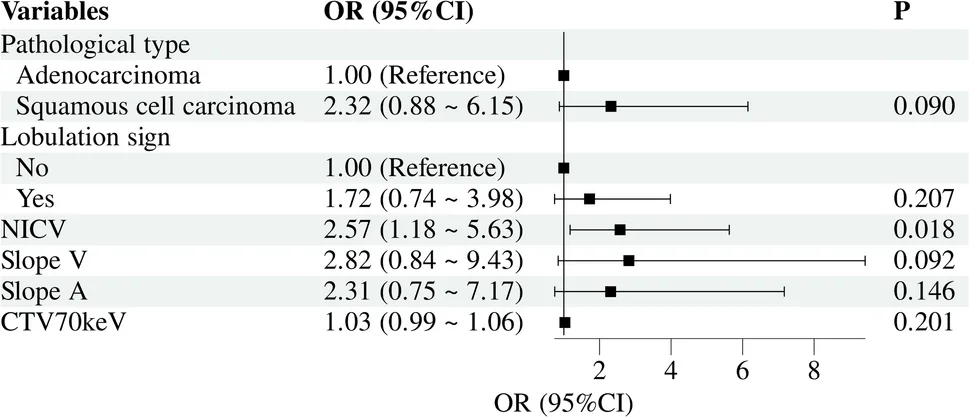
Forest plot of multivariate logistic regression analysis. Venous-phase normalized iodine concentration is identified as an independent predictive factor for epidermal growth factor receptor (EGFR) mutation (odds ratio [*OR*] = 2.578, 95% CI: 1.179–5.639, *P* = 0.018). Abbreviations: EGFR, epidermal growth factor receptor; OR, odds ratio; CI, confidence interval; NICV, venous-phase normalized iodine concentration; Slope A, arterial-phase spectral curve slope; Slope V, venous-phase spectral curve slope; CTV70keV, venous-phase 70-keV monoenergetic CT value

All variables with statistically significant differences (*P* < 0.05) in univariate analysis were included in the multivariate binary logistic regression model. Pathological type was included in the model as a covariate to adjust for potential confounding from histological heterogeneity. Multicollinearity test confirmed that the VIF values of all included variables ranged from 1.045 to 1.758, with no significant multicollinearity that would affect the stability of the model. Venous-phase NIC was identified as the only independent predictive factor for EGFR mutation (OR = 2.578, 95% CI: 1.179–5.639, *P* = 0.018) (Fig. 2).

### Comparison of predictive models

Two predictive models were constructed based on the multivariate regression results:

- Model 1 (clinical + conventional CT model): constructed with pathological type and lobulation sign.
- Model 2 (clinical + conventional CT + spectral CT combined model): constructed with all variables included in the regression. All continuous variables were entered into the model as raw measured values. Categorical variables were coded as follows: Pathological type (1=adenocarcinoma, 0=squamous carcinoma); Lobulation sign (1=present, 0=absent).

**Fig. 3:**
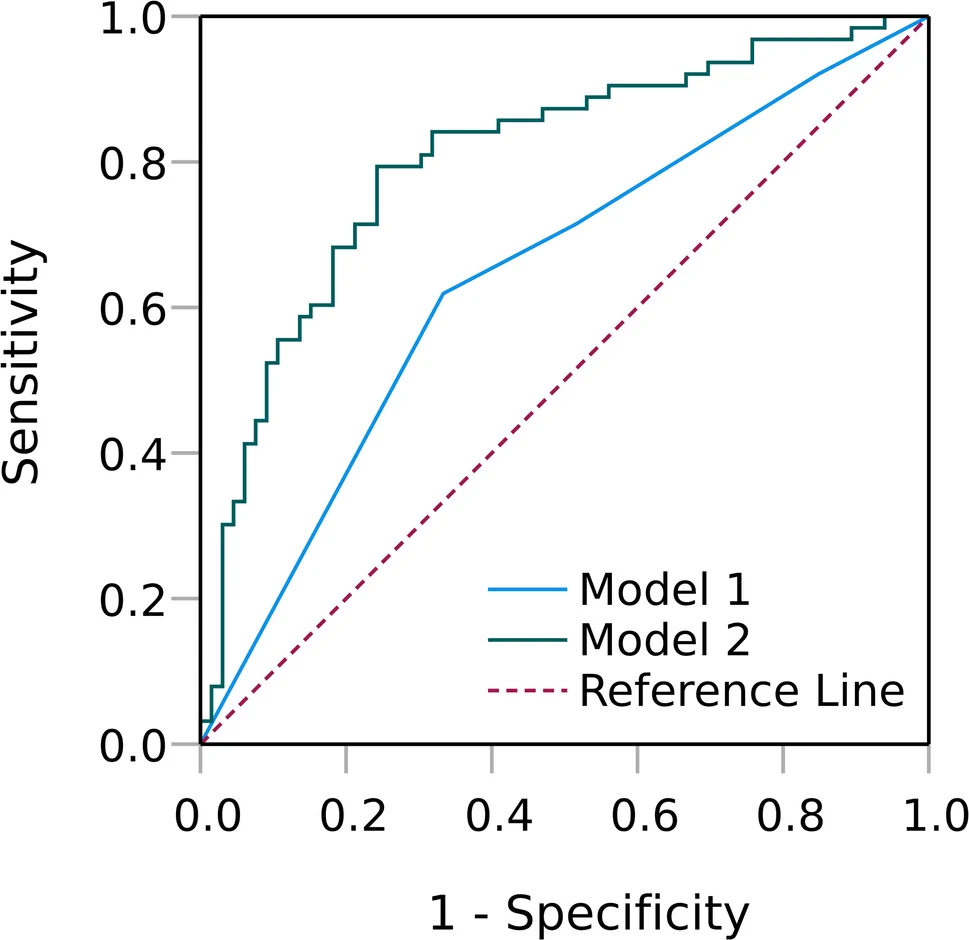
Comparison of receiver operating characteristic (ROC) curves between Model 1 (clinical + conventional CT) and Model 2 (clinical + conventional CT + spectral CT). The combined Model 2 shows significantly better predictive performance (area under the curve [AUC] = 0.805 vs. 0.642, *P* < 0.001, DeLong test). Abbreviations: ROC, receiver operating characteristic; CT, computed tomography; AUC, area under the curve

The combined Model 2 demonstrated significantly superior discriminative performance than the conventional Model 1 (DeLong test, *Z* = 3.522, *P* < 0.001) (Fig. 3). Internal validation via 1000-iteration bootstrap resampling confirmed the excellent robustness of the combined model. The regression coefficients of all predictors showed minimal deviation across resampling iterations, with a maximum absolute deviation of less than 0.11.The core predictor venous NIC remained an independent predictive factor for EGFR mutation after bootstrap validation (*P* = 0.010). The bootstrap-corrected AUC of the model was 0.792 (95% CI: 0.712–0.872), with a calibration slope of 0.947, indicating negligible overfitting and good generalization performance of the model.

The full performance metrics of the two models are summarized in Table 3. At the optimal cut-off value of 0.459, Model 2 achieved a sensitivity of 79.4%, a specificity of 75.8%, a PPV of 75.8%, an NPV of 79.4%, a PLR of 3.28, and an NLR of 0.27. The optimal cut-off value was determined based on our study cohort, and external validation in independent cohorts is required before widespread routine clinical application. Model 2 had a significantly lower Brier score than Model 1 (0.179 vs. 0.232), indicating higher prediction precision and lower systematic error. The Hosmer-Lemeshow test confirmed good calibration for both models (Model 1: *P* = 0.438; Model 2: *P* = 0.607).

**Table 3:** Full performance comparison of the two prediction models

Performance Metric	Model 1 (Clinical Conventional CT)	Model 2 (Combined Spectral CT Model)
AUC (95% CI)	0.642 (0.547–0.738)	0.805 (0.728–0.882)
Sensitivity (%)	61.0	79.4
Specificity (%)	66.7	75.8
Positive Predictive Value (%)	63.3	75.8
Negative Predictive Value (%)	63.8	79.4
Positive Likelihood Ratio	1.83	3.28
Negative Likelihood Ratio	0.58	0.27
Brier Score	0.232	0.179
Hosmer-Lemeshow *P*-value	0.438	0.607

*Abbreviations*: *AUC* area under the receiver operating characteristic curve, *CI* confidence interval, *CT* computed tomography. Model 1 was constructed with clinical features and conventional CT findings; Model 2 was the combined model integrating clinical features, conventional CT findings, and spectral CT quantitative parameters. 95% CIs of AUC were calculated using 1000-times bootstrap resampling

**Fig. 4:**
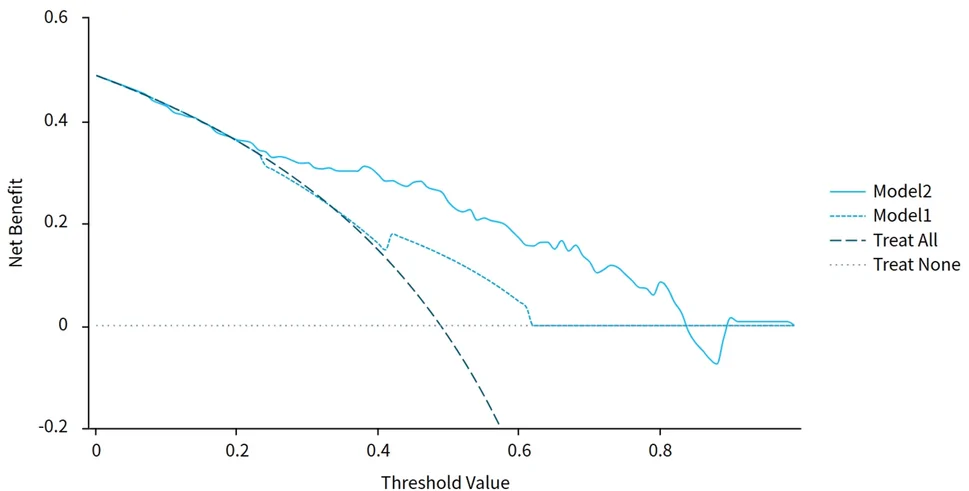
Decision curve analysis (DCA) for the two predictive models of classic epidermal growth factor receptor (EGFR)-activating mutations. The y-axis represents net benefit, and the x-axis represents the threshold probability of harboring classic EGFR-activating mutations. Model 2 (solid line): the combined spectral CT model integrating clinical features, conventional CT findings and dual-phase spectral CT parameters; Model 1 (long dashed line): the clinical-conventional CT model. The dash-dot line indicates the treat-all strategy, and the dotted line indicates the treat-none strategy. Across most clinically relevant threshold probabilities (0.1–0.9), Model 2 achieved a higher net benefit than Model 1 and the two default clinical strategies, confirming its superior clinical utility for preoperative patient stratification for EGFR tyrosine kinase inhibitor (TKI) therapy. Abbreviations: DCA, decision curve analysis; EGFR, epidermal growth factor receptor; CT, computed tomography; TKI, tyrosine kinase inhibitor

DCA further confirmed that Model 2 provided a higher net benefit than Model 1 and the treat-all/treat-none strategies across the majority of clinically relevant threshold probabilities (0.1–0.9). For the most commonly used clinical threshold range of 0.2–0.6, the net benefit of Model 2 ranged from 0.158 to 0.598, which was 2–3 times higher than that of Model 1, demonstrating superior clinical utility for preoperative selection of patients eligible for EGFR-TKI therapy (Fig. 4).

The final prediction equation for the combined Model 2 is as follows:

- Logit(P) = -7.525 + 0.846×Arterial-phase λHU - 0.840×Pathological type - 0.540×Lobulation sign + 0.947×Venous-phase NIC + 1.034×Venous-phase λHU + 0.025×Venous-phase 70-keV CT value.
- Note: Logit(P) = ln[P/(1-P)], where P is the predicted probability of EGFR mutation. This formula is derived from the internal validation of this study cohort, and further external validation in independent cohorts is required before widespread clinical routine application.

Variable assignment in the formula:

- Pathological type: 1 = adenocarcinoma, 0 = squamous cell carcinoma
- Lobulation sign: 1 = present, 0 = absent

All continuous variables were included as the original measured values.

In the pre-specified lung adenocarcinoma subgroup (*n* = 96, the core target population for EGFR-TKI therapy), the combined Model 2 still maintained excellent and stable predictive performance. The full performance metrics of the model in this subgroup are summarized in Table 4, with an AUC of 0.810 (95% CI: 0.722–0.898, *P* < 0.001). At the optimal cut-off value of 0.454, the model achieved a sensitivity of 82.7% and a specificity of 70.5%. This subgroup analysis was pre-specified and exploratory, and the results should be interpreted with caution before further validation in large-scale cohorts.

**Table 4:** Full performance of the combined model in the lung adenocarcinoma subgroup

Performance Metric	Value (95% CI)
AUC	0.810 (0.722–0.898)
Sensitivity (%)	82.7
Specificity (%)	70.5
Positive Predictive Value (%)	76.8
Negative Predictive Value (%)	77.4
Positive Likelihood Ratio	2.80
Negative Likelihood Ratio	0.24
Brier Score	0.176
Hosmer-Lemeshow *P*-value	0.736

*Abbreviations*: *AUC* area under the receiver operating characteristic curve, *CI* confidence interval, *CT* computed tomography. The combined model was constructed with clinical features, conventional CT findings, and spectral CT quantitative parameters. 95% CIs of AUC were calculated using 1000-times bootstrap resampling

## Discussion

In this study, we developed and internally validated a noninvasive spectral CT-based combined model for preoperative prediction of classic EGFR-activating mutations in NSCLC, which aligns with the core focus of clinical cancer research and personalized targeted therapy. While tissue-based genotyping remains the diagnostic gold standard for EGFR mutation detection, its invasive nature, risk of procedure-related complications, and long turnaround time have led to an unmet clinical need for noninvasive, readily implementable tools to streamline patient stratification for targeted therapy. Our combined model integrating clinical, conventional CT, and dual‑layer spectral CT parameters achieved significantly superior discriminative performance for predicting classic EGFR-activating mutations compared with a clinical-CT only model.

In single-parameter analysis, venous-phase λHU yielded the highest predictive efficacy (AUC = 0.741), followed by venous-phase NIC (AUC = 0.700, 95% CI: 0.611–0.789), consistent with previous dual-layer spectral CT studies demonstrating the association between venous-phase spectral parameters and EGFR mutation status [8, 15]. The biological basis for this association is well-established: EGFR mutations upregulate vascular endothelial growth factor (VEGF) expression, promoting tumor neovascularization and increasing microvessel density and vascular permeability [16]. These pathophysiological changes enhance tumor iodine uptake, which is directly quantified by spectral CT parameters including λHU and NIC. Notably, venous-phase parameters outperformed arterial-phase counterparts, which is consistent with findings from previous studies [8]. The improved stability of venous-phase measurements may be attributed to more consistent hemodynamic profiles and more homogeneous contrast distribution within tumor vasculature during the venous phase, which could reduce measurement variability associated with dynamic arterial inflow.

Multivariate logistic regression, which adjusts for potential confounders including pathological type and conventional CT features, identified venous-phase NIC as the only independent predictor of EGFR mutation. This finding complements our single-parameter results: while venous-phase λHU achieved the highest individual AUC, it lost statistical significance after adjusting for covariates, whereas NIC retained its predictive value. This finding is consistent with previous studies [8, 17], which also reported venous-phase NIC as a significant predictor of EGFR mutation status. Notably, NIC is normalized to aortic iodine concentration. This normalization eliminates confounding from individual differences in contrast dynamics and circulatory status, giving NIC superior stability and clinical applicability.

The combined model achieved an AUC of 0.805, significantly outperforming the clinical-CT only model (AUC = 0.642). This performance demonstrates the incremental value of incorporating spectral CT parameters into conventional clinical-CT workflows for preoperative EGFR mutation stratification. Importantly, the model offers direct and practical clinical translational value for guiding EGFR-TKI therapy, without intending to replace tissue genotyping, the current gold standard. To facilitate clinical translation, we propose the following integrated workflow scheme. For patients with pulmonary nodules or masses who are diagnosed or scheduled for radical resection, routine dual-phase dual-layer spectral CT enhancement has been integrated into the preoperative staging pathway in most clinical institutions. The spectral CT parameters, including Arterial-phase λHU, venous-phase NIC, venous-phase λHU, and venous-phase CT70keV are measured by radiologists on a standard spectral post-processing workstation following a predefined region of interest (ROI) protocol. The lobulation sign is extracted from routine CT reports, and the histopathological type is obtained from pathological diagnosis; the entire process requires no additional scanning time. These six variables are entered into a standalone prediction calculator (implementable via a departmental intranet mini-program or Excel spreadsheet) by radiologists or clinicians to generate the predicted EGFR mutation probability P. Using the study-validated optimal cut-off value of 0.459, patients with *P* ≥ 0.459 are prioritized for tissue-based EGFR genotyping. For patients with contraindications to invasive biopsy, the eligibility for EGFR-tyrosine kinase inhibitor therapy is evaluated via multidisciplinary team discussion. Patients with *P* < 0.459 are managed according to standard clinical workflows. This workflow does not replace tissue-based genotyping; instead, it serves as a triage auxiliary tool embedded within the existing preoperative CT pathway. Its core clinical application scenarios include the following three aspects:

First, for patients with contraindications to invasive biopsy (such as poor physical status, high-risk lesion location, or bleeding tendency), the model’s negative predictive value of 79.4% can help clinicians identify patients with low probability of EGFR mutation, avoiding unnecessary high-risk invasive procedures.

Second, for patients with resectable early-stage NSCLC, the model can provide preoperative prediction of EGFR mutation status, allowing the multidisciplinary team to proactively formulate neoadjuvant or adjuvant EGFR-TKI treatment plans, avoiding delays in targeted therapy initiation caused by the long turnaround time of postoperative genetic testing.

Third, for patients with wild-type results in single-site biopsy, the model can compensate for the limitation of tumor heterogeneity: patients with high mutation probability predicted by the model can be considered for repeat multi-site biopsy to avoid missing patients who may benefit from EGFR-TKIs.

The subgroup analysis further verified the model’s clinical applicability. In the lung adenocarcinoma subgroup, the core target population for EGFR-TKI therapy, the model maintained excellent predictive performance with an AUC of 0.810, slightly higher than the overall cohort. Accurate EGFR mutation prediction in this population is particularly critical, as it has the highest probability of benefiting from first-line EGFR-TKIs, and noninvasive tools can help clinicians efficiently identify high-benefit patients.

Currently, combination strategies involving EGFR tyrosine kinase inhibitors represent a highly promising approach to improving clinical outcomes in NSCLC patients. While TKI–anti-angiogenic regimens (e.g., erlotinib–bevacizumab) improved the progression-free survival in EGFR-mutant NSCLC [18], they are now an established standard rather than a recent development. EGFR-TKI plus immunotherapy aims to reduce residual tumor burden by engaging antitumor immunity, thereby enhancing the efficacy of third-generation EGFR-TKIs in NSCLC; however, current mainstream immune checkpoint inhibitors (ICIs) have shown limited synergy with EGFR-TKIs [19]. More recently, CD24 has been identified as a promising immunotherapeutic target to enhance the efficacy of third-generation EGFR-TKIs: third-generation EGFR-TKI exposure upregulates CD24 expression, a process mediated by the JNK–phospho-YY1(S247) signaling axis [20]. CD24 blockade (e.g., ATG-031, a novel anti-CD24 monoclonal antibody) exerts synergistic enhancement with third-generation EGFR-TKIs [20]. ATG-031 has demonstrated robust preclinical antitumor activity and is currently under Phase I evaluation [21]. As mutation-selective EGFR-TKI combinations evolve, accurate preoperative EGFR stratification will be increasingly critical. Although our dual-layer spectral CT–based model was developed in the single-agent EGFR‑TKI era, it holds promise as a noninvasive triage tool that can be integrated into combination‑era treatment workflows.

This study has two key strengths. First, we developed a multidimensional combined model that fully leverages the complementary value of clinical, conventional CT, and dual‑layer spectral CT data, achieving significantly better performance than the conventional model. The model strictly defined the mutation-positive group as patients with classic TKI-sensitive EGFR-activating mutations (exon 19 deletions and exon 21 L858R), directly screening candidates for approved EGFR-TKI therapy and enhancing its clinical relevance. Second, we implemented rigorous imaging quality control, including pre-specified inter-observer agreement assessment and standardized ROI placement protocols, ensuring reliable quantitative data; the model also maintained stable performance in the lung adenocarcinoma subgroup, the core population for EGFR-TKI therapy.

Several limitations of this study should be acknowledged. First, this is a single-center retrospective study with a moderate sample size of 129 patients, which may introduce selection bias. While 1000-iteration bootstrap internal validation confirmed the model’s robustness, multicenter external validation in larger, heterogeneous cohorts is still required to confirm generalizability across different institutions. A prospective multicenter validation study with standardized imaging and measurement protocols is currently in preparation. Second, molecular coverage was limited to classic EGFR-activating mutations, without systematic analysis of rare EGFR mutations or other common driver alterations. Rare subtypes may exhibit distinct imaging characteristics, and alternative driver mutations in the EGFR wild-type group may influence imaging phenotypes and cause misclassification, warranting future investigation. Third, the study was restricted to solid pulmonary lesions > 1 cm in diameter, excluding subsolid nodules dominated by ground-glass components and lesions ≤ 1 cm. This strategy mitigated the partial volume effect but left a notable clinical gap, as these nodule subtypes are highly prevalent in lung cancer screening populations. Future studies with dedicated scanning protocols are warranted to verify the model’s performance in these subgroups. Fourth, the model was developed and validated using data from a single Philips IQon dual‑layer spectral CT scanner. Cross-vendor harmonization of spectral parameters, including conversion equations derived from standardized physical phantoms and domain-adaptation approaches, will be required before widespread implementation. Finally, although all enrolled patients had available specimens for EGFR genotyping, patients who cannot undergo invasive biopsy—the core beneficiary population of this model—were not directly included. We will further verify the clinical value of the model in this population in our ongoing prospective multicenter study.

## Conclusions

In conclusion, the combined model integrating clinical features, conventional CT signs, and dual-phase spectral CT parameters developed in this study enables accurate, noninvasive preoperative prediction of classic EGFR-activating mutations in NSCLC patients. Venous-phase spectral curve slope achieved the highest single-parameter predictive value, while venous-phase normalized iodine concentration was identified as an independent predictor of EGFR mutation. Bootstrap internal validation confirmed the model’s excellent robustness and minimal overfitting. While not intended to replace tissue-based genotyping, this model provides a readily implementable, noninvasive adjunct to standard clinical workflows, facilitating optimized preoperative patient stratification for EGFR-TKI therapy and supporting personalized precision treatment decision-making for patients with NSCLC. Multicenter external validation of the model is currently in preparation.

## Supplementary Information

Supplementary Material 1.

## Data Availability

The datasets generated and analyzed during the current study are available from the corresponding authors on reasonable request.
